# Comparison of anchor screw fixation versus mini-plate fixation in unilateral expansive open-door laminoplasty for the treatment of multi-level cervical spondylotic myelopathy

**DOI:** 10.1097/MD.0000000000013534

**Published:** 2018-12-10

**Authors:** Xiang Lin, Kaiwei Chen, Haijun Tang, Xianying Huang, Changwu Wei, Zengming Xiao

**Affiliations:** a Department of Musculoskeletal Oncology, Affiliated Tumor Hospital of Guangxi Medical University; b Department of Spine Surgery, The First Affiliated Hospital of Guangxi Medical University, Nanning, China.

**Keywords:** anchor screw fixation, laminoplasty, meta-analysis, mini-plate fixation

## Abstract

**Study design:**

Systematic review and meta-analysis.

**Background:**

Anchor screw fixation and mini-plate fixation are widely used in unilateral open-door laminoplasty. There is a great controversy over the preferred fixation method. The purpose of this study is to evaluate the clinical outcomes between anchor screw fixation and mini-plate fixation for the treatment of multilevel cervical spondylotic myelopathy (MCSM).

**Methods:**

Related studies that compared the clinical effectiveness of anchor screw fixation and mini-plate fixation in cervical laminoplasty for the treatment of MCSM were acquired by a comprehensive search in PubMed, Embase, the Cochrane library, CNKI, VIP, and WANFANG up to March, 2018. Included studies were evaluated according to eligibility criteria. The main end points included: preoperative Japanese Orthopedic Association (JOA) scores, postoperative JOA scores, JOA scores improvement rate, preoperative and postoperative cervical range of motion (ROM), preoperative and postoperative cervical curvature index (CCI), lamina open angle, operation time, blood loss, C5 nerve palsy rate and axial symptoms rate.

**Results:**

Papers in English and Chinese were searched for the initial review, but only 12 articles in Chinese were included in this meta-analysis. All of the selected studies were of high quality as indicated by the Newcastle–Ottawa scale (NOS). Among 809 patients, 372 underwent anchor screw fixation and 437 underwent mini-plate fixation. The results of this meta-analysis indicated that no significant difference was found in preoperative JOA score, JOA scores improvement rate, preoperative CCI, preoperative ROM, C5 palsy rate and blood loss. However, compared with mini-plate fixation, anchor screw fixation patients showed higher axial symptoms rate [*RR* = 1.75, 95% *CI* (1.31, 2.35), P <.05], lower postoperative JOA scores [*SMD* = −0.38, 95% *CI* (−0.62, −0.15), P <.05], lower postoperative CCI [*SMD* = −0.64, 95% *CI* (−0.94, −0.33), P <.05], lower postoperative ROM [*SMD* = −1.11, 95% *CI* (−2.18, −0.04), P <.05], smaller lamina open angle [*SMD* = −1.98, 95% *CI* (−3.71, −0.24), P <.05], shorter operation time [*SMD* = −0.33, 95% *CI* (−0.59, −0.07), P <.05].

**Conclusions:**

Compared with anchor screw fixation, mini-plate fixation in cervical laminoplasty appears to achieve better clinical and radiographic outcomes with fewer surgical complications. However, future well-designed, randomized controlled trials are still needed to further confirm our results.

## 1 Introduction

Multilevel cervical spondylotic myelopathy (MCSM) usually leads to gradual deterioration of spinal cord dysfunction.^[1]^ A posterior surgical approach with unilateral open-door laminoplasty is the most common procedure for treating MCSM because of satisfactory clinical outcomes.^[2]^ Many techniques have been reported to fix the elevated lamina in open-door laminoplasty, such as traditional facet joint suturing, anchor screw fixation and mini-plate fixation.^[3]^ Mini-plate fixation has been considered better than traditional facet joint suturing technique based on published data.^[4,5]^ Currently, both the mini-plate fixation and anchor screw fixation techniques are widely applied in laminoplasty for treating MCSM, but the scientific support is weak as for which technique is superior.

The primary objective of this meta-analysis was to pool the published evidences to determine whether mini-plate fixation or anchor screw fixation was significantly better in clinical and radiographic outcomes, complications in patients with MCSM.

## 2 Materials and methods

### 2.1 Ethics statement

As all analyses were based on previously published studies, ethical approval was not necessary in this review.

### 2.2 Surgical technique

The surgical methods of the 2 fixation techniques are as follows: the median longitudinal incision of the cervical posterior approach was performed to expose the bilateral lamina and lateral mass of C3-7. Mini-plate fixation: after the lamina was opened, a titanium mini-plate was placed between the lamina and the lateral mass on the side of the door on C3-7. Anchor screw fixation: on the hinge side, a 12 mm anchor screw with double-suture lines was placed on C3-7 lateral mass. After the lamina door was opened, the suture line was tightened and tied firmly through the spinal process to anchor the lamina.

### 2.3 Search strategy and study selection

We searched for studies published up to March 2018 that compared clinical effectiveness of mini-plate fixation and anchor screw fixation in cervical laminoplasty for the treatment of MCSM. The databases included PubMed, Embase, the Cochrane library, CNKI (Chinese database), VIP (Chinese database), and WANFANG (Chinese database). The languages were restricted to Chinese or English and only the published articles were included. The following search terms were used:

(1) cervical spondylotic myelopathy or CSM or ossification of posterior longitudinal ligament or OPLL;
(2) unilateral or single or open door or laminoplasty;
(3) anchor screw or anchoring fixation or screw;
(4) mini plate or microplate or plate fixation; (1) and (2) and (3) and (4) Reference lists of all included studies were scanned to identify additional potentially relevant studies. Two reviewers independently screened the titles and abstracts of identified papers, and full-text copies of all potentially relevant studies were obtained.

### 2.4 Inclusion criteria

Studies were included if they met the following criteria:

(1) study design: randomized or non-randomized controlled studies or and cohort studies;
(2) study population: patients with MCSM;
(3) purpose of interventions: to compare clinical outcomes difference between mini-plate fixation and anchor screw fixation in cervical laminoplasty;
(4) outcome measurements: at least 1 desirable outcome that means eligible and resultant variable. Studies did not meet the above criteria were excluded from selection.

### 2.5 Quality assessment of included studies

The Newcastle–Ottawa quality assessment scale (NOS) was used to evaluate the quality of the included studies.

### 2.6 Data extraction

The following information was extracted from each study:

(1) basic characteristics, including publication year, study design, patient age, enrolled number and follow-up time;
(2) primary outcome presented as preoperative JOA scores, postoperative JOA scores, JOA scores improvement rate, preoperative and postoperative ROM, preoperative and postoperative CCI, lamina open angle, C5 nerve palsy rate, and axial symptoms rate.
(3) secondary outcomes, including operation time, blood loss.

### 2.7 Data analysis

We performed all meta-analyses with the Review Manager software (RevMan Version 5.3, The Nordic Cochrane Center, The Cochrane Collaboration, Copenhagen, Denmark). Heterogeneity was tested using Chi-square test and quantified by calculating I^2^ statistic, for which *P* <.1 and I^2^ > 50% was considered to be statistically significant. For the pooled effects, standardized mean difference (SMD) was calculated for continuous variables and risk ratio (RR) was calculated for dichotomous variables. Continuous variables are presented as SMDs and 95% confidence intervals (CI), whereas dichotomous variables are presented as RRs and 95% CI. Random-effects or fixed-effects models were used depending on the heterogeneity of the studies included.

## 3 Results

### 3.1 Search results and quality assessment

**Figure 1:**
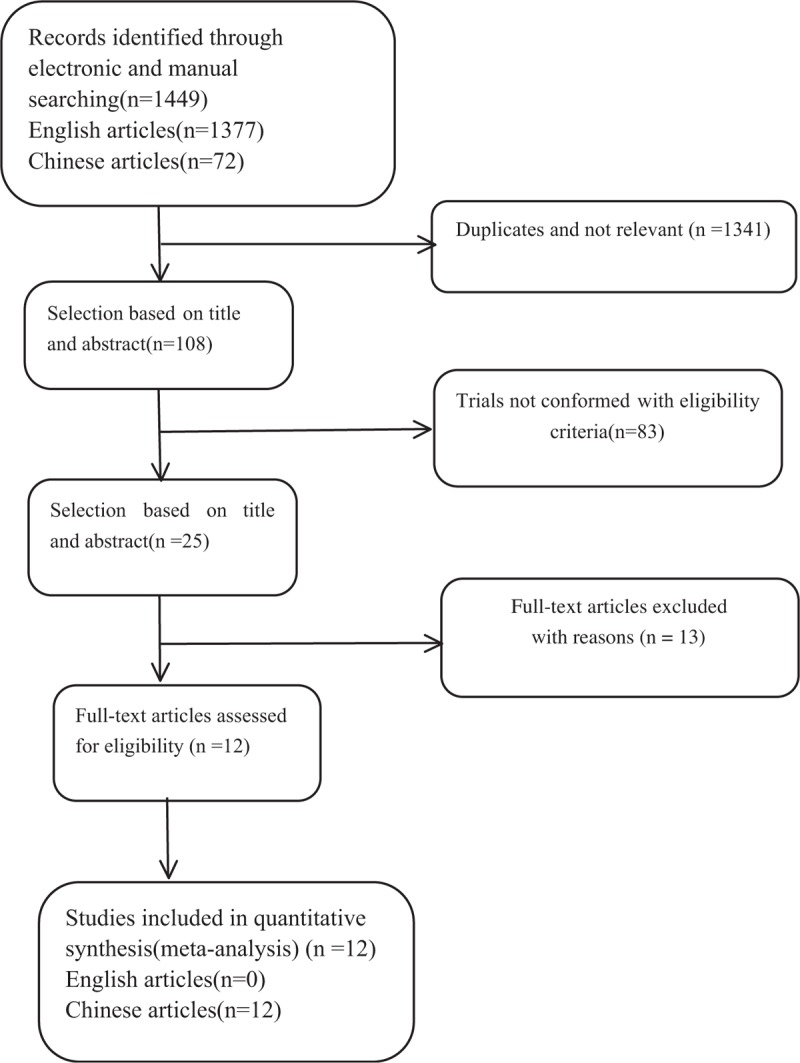
The flow chart shows the article selection process.

The process of identifying relevant studies is summarized in Figure 1. From the selected databases, 1449 references were obtained. By screening the titles and abstracts, 1424 references were excluded due to duplicates, irrelevant studies, case reports, not comparative studies and review. The remaining 25 reports underwent a detailed and comprehensive evaluation. 2 systematic reviews or meta-analysis were not eligible because of lack of primary data. 4 studies were ruled out because of comparisons with laminectomy with fusion; 3 studies were excluded because the patients received instrumented fusion; 3 studies were excluded because patients underwent only mini-plate fixation; 1 studies was ruled out because it did not give available data related to MCSM patients. Finally, 12 studies were included in this meta-analysis.^[6–17]^ All of the 12 studies were published in Chinese. Table 1 and Table 2 summarize the baseline characteristics assessment and quality of included studies, respectively. As all studies included were non-randomized controlled studies, the NOS was used to assess the quality of each study. All studies scored from 7 to 8 points, so the quality of each study was relatively high.

**Table 1:**
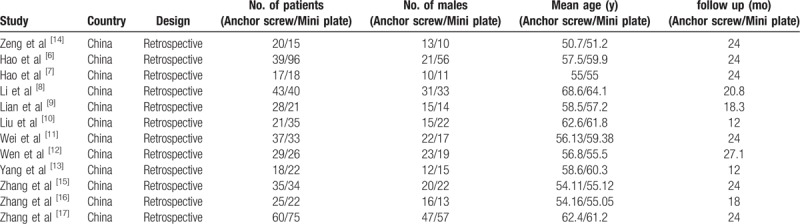
Characteristics of included studies.

**Table 2:**
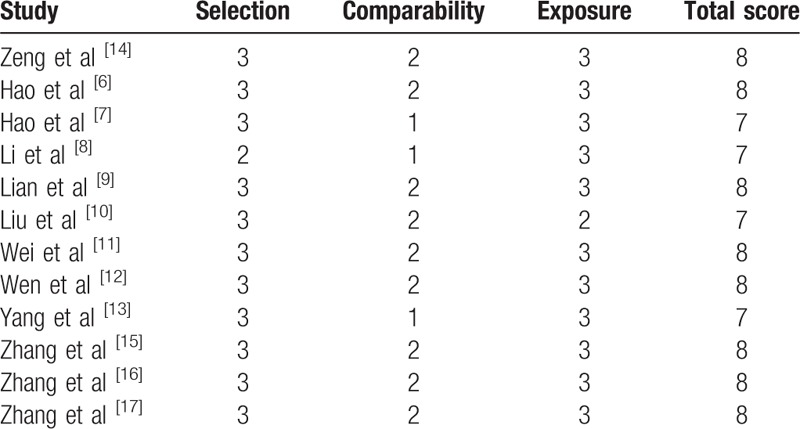
Quality assessment of included studies according to Newcastle–Ottawa scale (NOS).

### 3.2 Clinical evaluation

#### 3.2.1 Preoperative JOA scores

**Figure 2:**
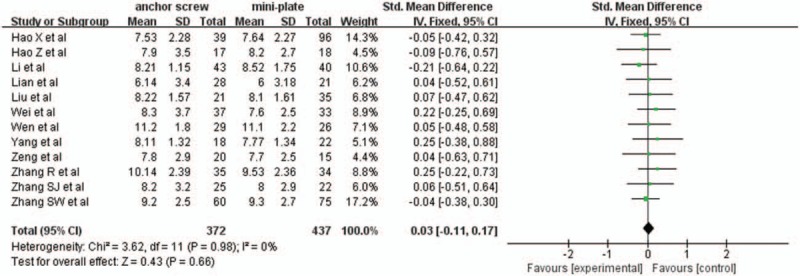
Forest plot of preoperative JOA scores between anchor screw group and mini-plate group. JOA = Japanese Orthopedic Association.

Twelve studies with a total of 809 patients, 372 (45.9%) in anchor screw group and 437 (54.0%) in mini-plate group, provided preoperative JOA scores. The research had no statistically significant heterogeneity (*P* = .98, I^2^ = 0%), fixed effect model was used as the pooling method, SMD was applied to analysis overall effect. There was no statistically significant difference in preoperative JOA scores between anchor screw group and mini-plate group [SMD = 0.03, 95%CI: −0.11, 0.17; *P* = .66; Fig. 2].

#### 3.2.2 Postoperative JOA scores

**Figure 3:**
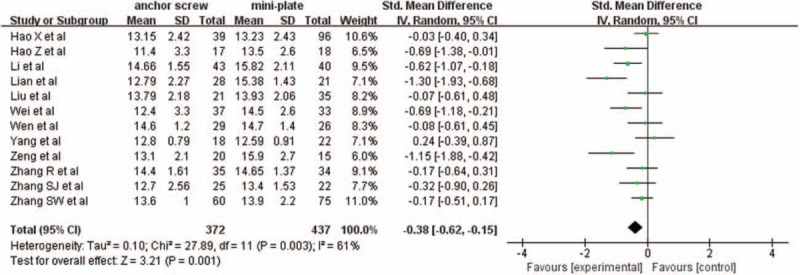
Forest plot of postoperative JOA scores between anchor screw group and mini-plate group. JOA = Japanese Orthopedic Association.

Twelve studies with a total of 809 patients, 372 (45.9%) in anchor screw group and 437 (54.0%) in mini-plate group, provided postoperative JOA scores. The research had statistically significant heterogeneity (*P* = .003, I^2^ = 61%), random effect model was used as the pooling method, SMD was applied to analysis overall effect. The postoperative JOA scores were significant lower in anchor screw group compared with mini-plate group [SMD = −0.38, 95% CI: −0.62, −0.15; *P* = .001; Fig. 3].

#### 3.2.3 JOA scores improvement rate

**Figure 4:**
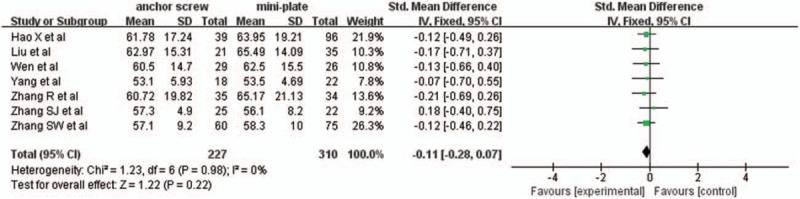
Forest plot of JOA scores improvement rate between anchor screw group and mini-plate group. JOA = Japanese Orthopedic Association.

Seven studies with a total of 537 patients, 227 (42.3%) in anchor screw group and 310 (57.7%) in mini-plate group, provided JOA scores improvement rate. The research had no statistically significant heterogeneity (*P* = .98, I^2^ = 0%), fixed effect model was used as the pooling method, SMD was applied to analysis overall effect. There was no statistically significant difference in JOA scores improvement rate between anchor screw group and mini-plate group [SMD = −0.11, 95% CI: −0.28, 0.07; *P* = .22; Fig. 4].

#### 3.2.4 Preoperative CCI

**Figure 5:**
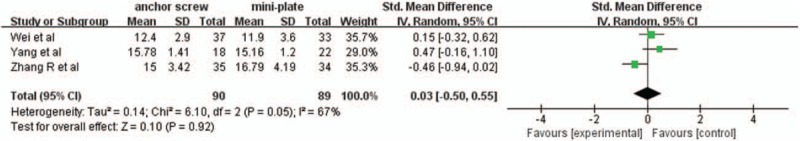
Forest plot of preoperative CCI between anchor screw group and mini-plate group. CCI = cervical curvature index.

Three studies with a total of 179 patients, 90 (50.3%) in anchor screw group and 89 (49.7%) in mini-plate group, provided preoperative CCI. The research had statistically significant heterogeneity (*P* = .05, I^2^ = 67%), random effect model was used as the pooling method, SMD was applied to analysis overall effect. There was no statistically significant difference in preoperative CCI between anchor screw group and mini-plate group [SMD = 0.03, 95% CI: −0.50, 0.55; *P* = .92; Fig. 5].

#### 3.2.5 Postoperative CCI

**Figure 6:**
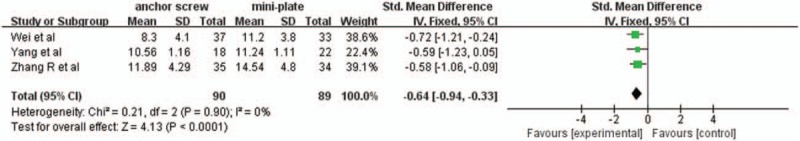
Forest plot of postoperative CCI between anchor screw group and mini-plate group. CCI = cervical curvature index.

Three studies with a total of 179 patients, 90 (50.3%) in anchor screw group and 89 (49.7%) in mini-plate group, provided postoperative CCI. The research had no statistically significant heterogeneity (*P* = .90, I^2^ = 0%), fixed effect model was used as the pooling method, SMD was applied to analysis overall effect. The postoperative CCI was significantly lower in anchor screw group compared with mini-plate group [SMD = −0.64, 95% CI: −0.94,−0.33; *P* < .0001; Fig. 6].

#### 3.2.6 Preoperative ROM

**Figure 7:**
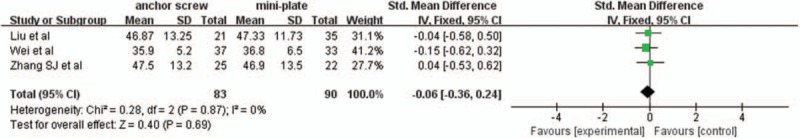
Forest plot of preoperative ROM between anchor screw group and mini-plate group. ROM = range of motion.

Three studies with a total of 173 patients, 83 (48.0%) in anchor screw group and 90 (52.0%) in mini-plate group, provided preoperative ROM. The research had no statistically significant heterogeneity (*P* = .87, I^2^ = 0%), fixed effect model was used as the pooling method, SMD was applied to analysis overall effect. There was no statistically significant difference in preoperative ROM between anchor screw group and mini-plate group [SMD = −0.06, 95% CI: −0.36, 0.24; *P* = .69; Fig. 7].

#### 3.2.7 Postoperative ROM

**Figure 8:**
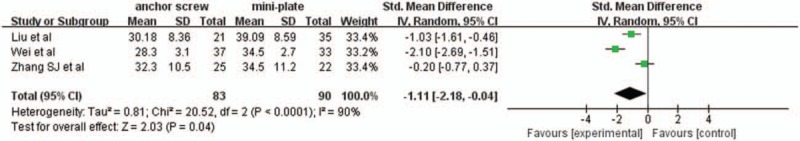
Forest plot of postoperative ROM between anchor screw group and mini-plate group. ROM = range of motion.

Three studies with a total of 173 patients, 83 (48.0%) in anchor screw group and 90 (52.0%) in mini-plate group, provided postoperative ROM. The research had statistically significant heterogeneity (*P* < .0001, I^2^ = 90%), random effect model was used as the pooling method, SMD was applied to analysis overall effect. The postoperative ROM was significantly lower in anchor screw group compared with mini-plate group [SMD = −1.11, 95% CI: −2.18, −0.04; *P* = .04; Fig. 8].

#### 3.2.8 Lamina open angle

**Figure 9:**
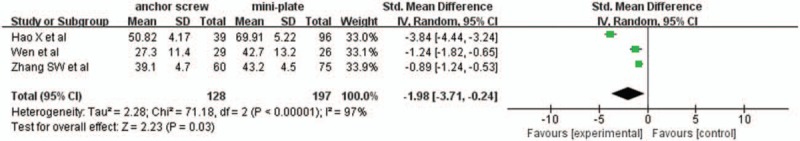
Forest plot of lamina open angle between anchor screw group and mini-plate group.

Three studies with a total of 325 patients, 128 (39.4%) in anchor screw group and 197 (60.6%) in mini-plate group, provided lamina open angle. The research had statistically significant heterogeneity (*P* <.00001, I^2^ = 97%), random effect model was used as the pooling method, SMD was applied to analysis overall effect. The lamina open angle was significantly smaller in anchor screw group compared with mini-plate group [SMD = −1.98, 95% CI: −3.71, −0.24; *P* = .03; Fig. 9].

#### 3.2.9 C5 nerve palsy rate

**Figure 10:**
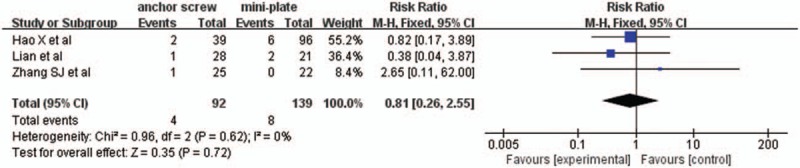
Forest plot of C5 nerve palsy rate between anchor screw group and mini-plate group.

Three studies with a total of 231 patients, 92 (39.8%) in anchor screw group and 139 (60.2%) in mini-plate group, provided C5 nerve palsy rate. The research had no statistically significant heterogeneity (*P* = .62, I^2^ = 0%), fixed effect model was used as the pooling method, RR was applied to analysis overall effect. There was no statistically significant difference in C5 nerve palsy rate between anchor screw group and mini-plate group [RR = 0.81, 95% CI: 0.26, 2.55; *P* = .72; Fig. 10].

#### 3.2.10 Axial symptoms rate

**Figure 11:**
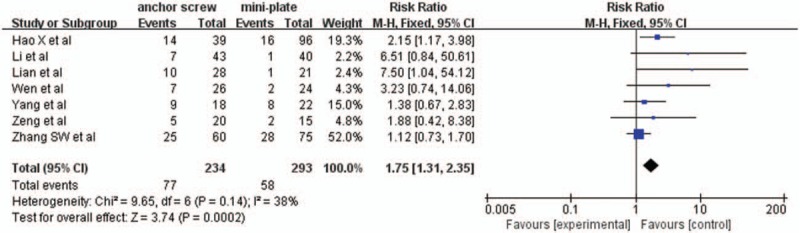
Forest plot of axial symptoms rate between anchor screw group and mini-plate group.

Seven studies with a total of 527 patients, 234 (44.4%) in anchor screw group and 293 (55.6%) in mini-plate group, provided axial symptoms rate. The research had no statistically significant heterogeneity (*P* = .14, I^2^ = 38%), fixed effect model was used as the pooling method, RR was applied to analysis overall effect. The axial symptoms rate was significantly higher in anchor screw group compared with mini-plate group [RR = 1.75, 95% CI: 1.31, 2.35; *P* = .0002; Fig. 11].

#### 3.2.11 Operation time

**Figure 12:**
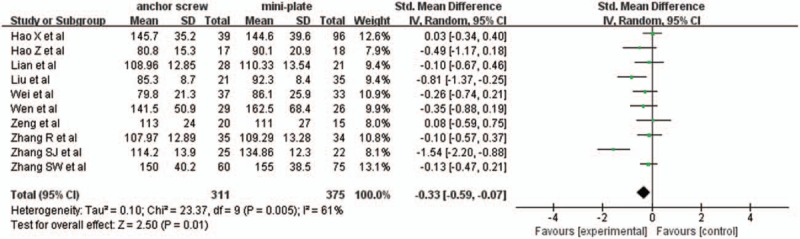
Forest plot of operation time between anchor screw group and mini-plate group.

Ten studies with a total of 686 patients, 311 (45.3%) in anchor screw group and 375 (54.7%) in mini-plate group, provided operation time. The research had statistically significant heterogeneity (*P* = .005, I^2^ = 61%), random effect model was used as the pooling method, SMD was applied to analysis overall effect. The operation time was significantly shorter in anchor screw group compared with mini-plate group [SMD = −0.33, 95% CI: −0.59, −0.07; *P* = .01; Fig. 12].

#### 3.2.12 Blood loss

**Figure 13:**
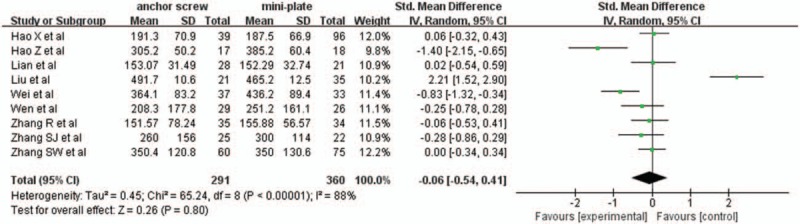
Forest plot of blood loss between anchor screw group and mini-plate group.

Nine studies with a total of 651 patients, 291 (44.7%) in anchor screw group and 360 (55.3%) in mini-plate group, provided blood loss. The research had statistically significant heterogeneity (*P* <.00001, I^2^ = 88%), random effect model was used as the pooling method, SMD was applied to analysis overall effect. There was no statistically significant difference in blood loss between anchor screw group and mini-plate group [SMD = −0.06, 95% CI: −0.54, 0.41; *P* = .80; Fig. 13].

Sensitivity analysis Sensitivity analysis was performed to confirm the stability of this meta-analysis by sequentially omitting individual eligible studies. The pooled results were not significantly changed after each study was excluded, which showed the stability of the results.

**Figure 14:**
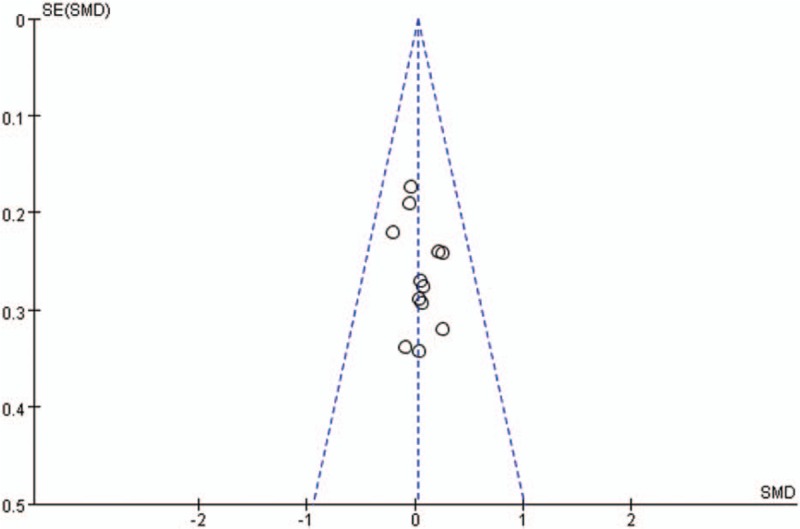
Funnel plots for preoperative JOA scores. JOA = Japanese Orthopedic Association.

**Figure 15:**
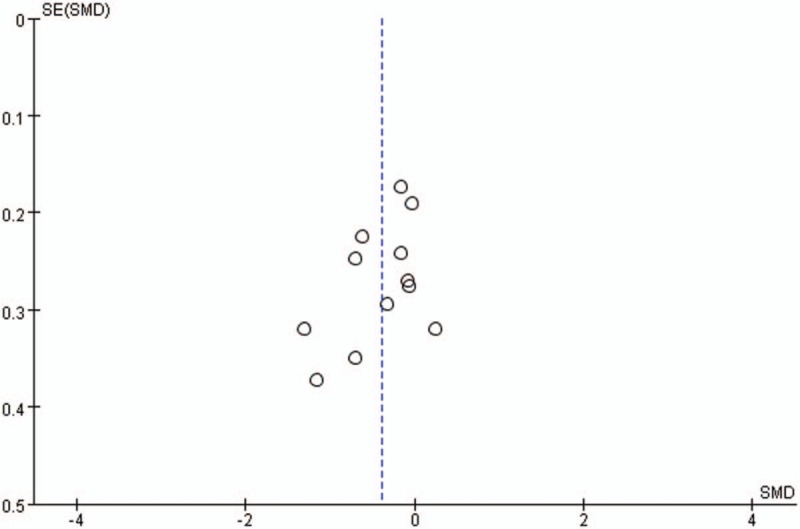
Funnel plots for postoperative JOA scores. JOA = Japanese Orthopedic Association.

**Figure 16:**
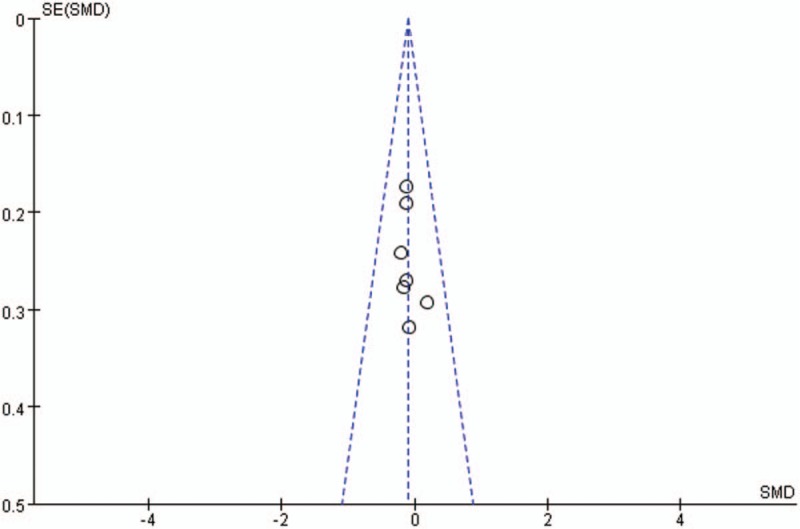
Funnel plots for JOA scores improvement rate. JOA = Japanese Orthopedic Association.

**Figure 17:**
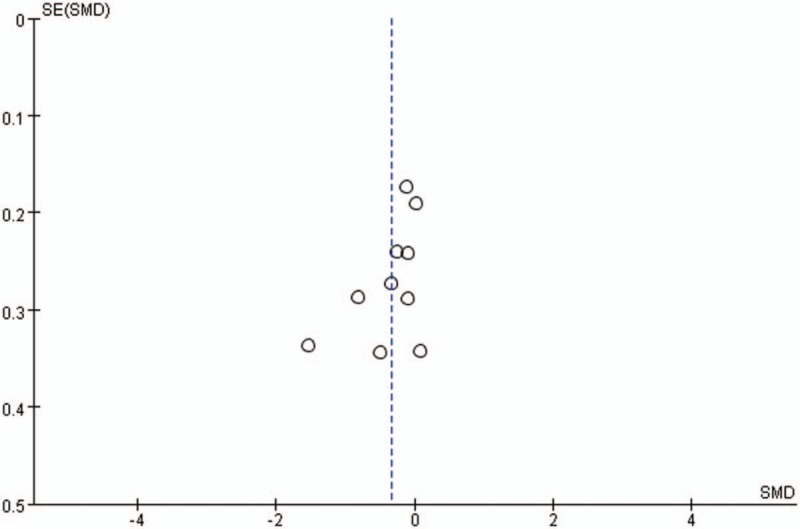
Funnel plots for operation time.

Publication bias for included studies was assessed by funnel plots (Figs. 14–17). Funnel plots showed nearly symmetric for preoperative JOA scores, postoperative JOA scores, JOA scores improvement rate and operation time, indicating no significant publication bias among the included studies.

## 4 Discussion

Cervical laminoplasty can provide satisfactory clinical outcomes in treating MCSM by expansive decompression of the spinal cord.^[18]^ Traditionally, the opened lamina are fixed by classical suture suspension method, but suture suspension cannot provide a sufficient rigid fixation. In recent years, alternative fixation techniques such as mini-plate fixation and anchor screw fixation are widely used in cervical laminoplasty. It has not been confirmed that which technique is superior. Zeng et al^[14]^ reported that laminoplasty with mini-plate fixation showed better postoperative JOA scores and fewer surgical complications. Hao et al^[6]^ reported that mini-plate fixation preserved more cervical ROM and provided lower axial symptoms rate, but there were no significant differences in postoperative JOA scores between 2 groups. Wei et al^[11]^ reported that mini-plate fixation obtained better postoperative JOA scores but more operation time and blood loss compared with anchor screw fixation.

In this meta-analysis, we combined 12 studies that included a total of 437 (54.0%) patients in mini-plate group and 372 (45.9%) patients in anchor screw group. Compared with anchor screw fixation in cervical laminoplasty, mini-plate fixation showed better clinical outcomes and fewer surgical complications, but with more operation time.

JOA scores are widely applied to assess clinical outcomes. The pooled data showed that there was no statistically significant difference in preoperative JOA scores and JOA scores improvement rate between 2 groups. However, there was statistically significant difference in postoperative JOA scores between 2 groups that indicated mini-plate fixation was superior to anchor screw fixation in improving clinical outcomes.

ROM and CCI were selected for analysis. The pooled data showed that there was no statistically significant difference in preoperative ROM and CCI between 2 groups. However, there were statistically significant differences in postoperative ROM and CCI between 2 groups, which indicated mini-plate fixation was superior to anchor screw fixation in preserving cervical ROM and cervical alignment. The reasons may be mini-plate fixation is able to offer an instant rigid fixation for the opened lamina with quick functional rehabilitation exercise while anchor screw fixation needs to immobilize the patients for even more time that can result in cervical back muscle atrophy.^[6,10,12,13,19]^

The postoperative lamina open angle was often selected to assess the drift of the spinal cord and the effect of the spinal canal decompression.^[20]^ The pooled data showed that there was statistically significant difference in postoperative lamina open angle between 2 groups that indicated mini-plate fixation was superior to anchor screw fixation in the drift of the spinal cord and the effect of the spinal canal decompression. The cause might be that compared with anchor screw fixation, mini-plate fixation can offer an immediately rigid fixation of the opened lamina while preventing lamina re-closure to get greater drift of the spinal cord.^[6,12]^

Axial symptoms and C5 palsy were selected for analysis to evaluate postoperative complications. The pooled data showed that there was no statistically significant difference in C5 palsy between 2 groups. However, there was statistically significant difference in axial symptoms between 2 groups, which indicated mini-plate fixation was superior to anchor screw fixation in reducing the incidence of axial symptoms. mini-plate fixation can provide an instant rigid fixation for the opened lamina with quick functional rehabilitation exercise while anchor screw fixation needs to immobilize the patients for even more time that can cause cervical back muscle atrophy, which may also result in axial symptoms.^[21,22]^

Operation time and blood loss were very important aspects for evaluating surgical injury. The pooled data showed that there was no statistically significant difference in blood loss between 2 groups. However, there was statistically significant difference in operation time between 2 groups, which indicated mini-plate fixation was associated with greater surgical injury. For older patients with underlying diseases, anchor screw fixation may be suitable.^[11]^

We believe that the results of this meta-analysis are affected by several reasons. First, all of the included studies are not randomized controlled trials in this meta-analysis. Second, there was variability choosing indicators to evaluate the clinical outcomes between the included studies, indicating the lack of standard outcome measurements. Third, the length of follow-up varied between studies and this is important for surgical outcomes evaluation. Finally, clinical heterogeneity might be caused by the various indications for operations.

## 5 Conclusions

Compared with anchor screw fixation in cervical laminoplasty, mini-plate fixation appears to provide better clinical and radiographic outcomes with fewer surgical complications. The level of evidence is III and the grade of recommendation is B according to the Evidence-Based Guidelines of the North American Spine Society (NASS).^[23]^ However, future well-designed, randomized controlled trials are still needed to further confirm our results.

## Author contributions

**Conceptualization:** Xiang Lin, Kaiwei Chen, Changwu Wei, Zengming Xiao.

**Data curation:** Xiang Lin, Haijun Tang, Xianying Huang, Changwu Wei.

**Formal analysis:** Xiang Lin, Kaiwei Chen, Zengming Xiao.

**Funding acquisition:** Changwu Wei, Zengming Xiao.

**Investigation:** Xiang Lin, Kaiwei Chen.

**Methodology:** Xiang Lin, Kaiwei Chen, Haijun Tang, Zengming Xiao.

**Project administration:** Xiang Lin, Xianying Huang, Zengming Xiao.

**Resources:** Haijun Tang.

**Software:** Xiang Lin, Kaiwei Chen.

**Supervision:** Zengming Xiao.

**Validation:** Xiang Lin, Haijun Tang, Xianying Huang, Changwu Wei, Zengming Xiao.

**Visualization:** Xianying Huang.

**Writing – original draft:** Xiang Lin.

**Writing – review & editing:** Xiang Lin, Zengming Xiao.
